# Impaired training-induced angiogenesis process with loss of pericyte-endothelium interactions is associated with an abnormal capillary remodelling in the skeletal muscle of COPD patients

**DOI:** 10.1186/s12931-019-1240-6

**Published:** 2019-12-05

**Authors:** Léo Blervaque, Emilie Passerieux, Pascal Pomiès, Matthias Catteau, Nelly Héraud, Marine Blaquière, François Bughin, Bronia Ayoub, Nicolas Molinari, Jean-Paul Cristol, Antonia Perez-Martin, Jacques Mercier, Maurice Hayot, Fares Gouzi

**Affiliations:** 1 Physiologie & médecine expérimentale du Cœur et des Muscles (PhyMedExp), INSERM - CNRS - Montpellier University. CHU Arnaud De Villeneuve, 371 avenue du Doyen Gaston Giraud, 34295 Montpellier cedex 5, France; 2 Les Cliniques du Souffle®, Groupe 5 Santé, Lodève, France; 3 PhyMedExp, INSERM – CNRS, Montpellier University, CHU Montpellier, Montpellier, France; 4 IMAG, CNRS, Montpellier University, CHU Montpellier, Montpellier, France; 5 Vascular Medicine Department and Laboratory, CHU Nîmes and EA2992 Research Unit, Montpellier University, Nimes, France

**Keywords:** COPD, Capillaries, Angiogenesis, Skeletal muscle, Exercise training

## Abstract

**Abstract:**

Chronic obstructive pulmonary disease (COPD) is associated with exercise intolerance and limits the functional gains in response to exercise training in patients compared to sedentary healthy subjects (SHS). The blunted skeletal muscle angiogenesis previously observed in COPD patients has been linked to these limited functional improvements, but its underlying mechanisms, as well as the potential role of oxidative stress, remain poorly understood. Therefore, we compared ultrastructural indexes of angiogenic process and capillary remodelling by transmission electron microscopy in 9 COPD patients and 7 SHS after 6 weeks of individualized moderate-intensity endurance training. We also assessed oxidative stress by plasma-free and esterified isoprostane (F_2_-IsoP) levels in both groups. We observed a capillary basement membrane thickening in COPD patients only (*p* = 0.008) and abnormal variations of endothelial nucleus density in response to exercise training in these patients when compared to SHS (*p* = 0.042). COPD patients had significantly fewer occurrences of pericyte/endothelium interdigitations, a morphologic marker of capillary maturation, than SHS (*p* = 0.014), and significantly higher levels of F_2_-IsoP (*p* = 0.048). Last, the changes in pericyte/endothelium interdigitations and F_2_-IsoP levels in response to exercise training were negatively correlated (r = − 0.62, *p* = 0.025). This study is the first to show abnormal capillary remodelling and to reveal impairments during the whole process of angiogenesis (capillary creation and maturation) in COPD patients.

**Trial registration:**

NCT01183039 & NCT01183052, both registered 7 August 2010 (retrospectively registered).

## Background

Chronic obstructive pulmonary disease (COPD) is characterized by a persistent airflow limitation due to airway and/or alveolar abnormalities, and it is systematically associated with other concomitant chronic diseases [1]. Limb skeletal muscle dysfunction, for example, affects the morbi-mortality of these patients [2–4]. Yet, skeletal muscle capillarization, despite its key role in this dysfunction in COPD patients [5], is an under-considered explanation of this phenomenon. A previous study of our group nevertheless demonstrated a significantly lower increase in skeletal muscle capillarization in response to exercise training in COPD patients compared with sedentary healthy subjects (SHS) [6]. This blunted angiogenesis was linked to the limited functional gains from exercise training observed in the patients [6]. Thus, while impaired angiogenesis appears to be relevant to the muscle misadaptations to exercise training in COPD patients, it remains insufficiently understood.

Physiologically, the angiogenic process in response to exercise training is highly dynamic, resulting from a balance between pro- and anti-angiogenic factors. However, two main phases of angiogenesis have been described: an early phase of new capillary creation and a later phase of neo-capillary maturation [7]. The morphologic markers of capillary creation [abluminal endothelial (EC) sprouts or intraluminal EC folding] and maturation [pericyte/endothelium (Pc/EC) interdigitations] in skeletal muscle were initially described in studies using transmission electron microscopy (TEM) [8–11], which is the reference method for their identification [12, 13]. Capillary remodelling in response to exercise training also appears to be linked to angiogenesis [12], and it too can be assessed by TEM through a morphometric analysis of the main capillary components (lumen, endothelium, basement membrane and pericytes) [8].

In chronic diseases like diabetes, both impaired angiogenesis [14] and pathological capillary remodelling [15] have been described in response to exercise training. Oxidative stress is a potential factor of these impairments, causing basement membrane protein accumulation [16] and pericyte apoptosis [17]. Moreover, oxidative stress promotes isoprostane formation, [18] which is a direct inhibitor of angiogenesis [19] and a promotor of pericyte loss [20]. In COPD patients, increased levels of plasma-free and esterified isoprostanes (F_2_-IsoP) and muscle oxidative stress have been reported [5, 21, 22]. We thus hypothesized that the blunted angiogenesis in COPD patients in response to exercise training [6] would be associated with abnormal capillary remodelling, an impaired angiogenic process (i.e. capillary creation and maturation), and increased plasmatic oxidative stress levels.

Therefore, the aim of this study was to compare capillary remodelling and the morphologic markers of the angiogenic process in skeletal muscle after a 6-week exercise training programme in COPD patients and SHS. In addition, we sought to determine whether the changes in the capillary ultrastructure and the changes in plasmatic levels of F_2_-IsoP were correlated in both groups.

## Materials and methods

### Study design

This study was conducted on skeletal muscle samples from subgroups of both COPD patients and SHS recruited in two previous studies (registration numbers for clinicaltrial.gov: NCT01183039 and NCT01183052). The subjects were recruited at PhyMedExp (INSERM-CNRS-UM), Montpellier University Hospital, Montpellier, France, or the “La Solane” and “La Vallonie” Pulmonary Rehabilitation Centres in Osseja and Lodève, France. These studies were the subject of a previous publication [Gouzi et al., 2013] [6]. The present study used TEM to more closely investigate the underlying mechanisms of the blunted angiogenesis in response to exercise training found in the main study. The inclusion criteria are available in the previous study [6] and in clinicaltrials.gov/. Briefly, COPD patients were defined by dyspnoea, chronic cough or sputum production and/or a history of exposure to risk factors for the disease, and post-bronchodilator forced expiratory volume in 1 s/forced ventilator capacity ratio < 70% [23]. The exclusion criteria were other respiratory diagnoses, decompensated comorbidity, exacerbation within the last 3 months, and previous participation in an exercise training programme. Study protocols followed the European guidelines for good clinical practice and the Declaration of Helsinki.

### Pulmonary function assessment

Pulmonary function was assessed using whole-body plethysmography (Transmural Bodybox 2800; Sensomedics, Yorba Linda, CA, USA). PaO_2_ was measured in radial artery blood samples from seated COPD patients under room air condition, with a blood gas analyser (Roche OMNIS, Roche Diagnostics, Mannheim, Germany).

### Assessment of skeletal muscle function and exercise

The 6-min walking test was conducted in accordance with the ATS statement [24]. COPD patients and SHS also performed an incremental cycloergometric test on a calibrated, electrically-braked cycloergometer (Ergoselect 200P, Ergolyne, Bitz, Germany) in accordance with the ATS/ACCP statement [25]. The mean $\mathrm{V}\dot {\mathrm{O}}_2$ value over the last 20 s of the test was used as symptom-limited oxygen uptake ($\mathrm{V}\dot {\mathrm{O}}_{\mathrm{sl}}$). The endurance time of the quadriceps (Tlim_quad_) was assessed at a frequency of 10 movements per minute at 30% of the maximal voluntary contraction, as previously described [6]. The level of physical activity was assessed using the Voorrips questionnaire (modified Baecke’s questionnaire) [26].

### Exercise training programme

The exercise training programme was conducted for 6 weeks with 3 or 4 sessions per week (20 sessions in total) and was supervised by an experienced clinician. The protocol was consistent with the ATS/ERS statements [27]. The sessions lasted 1.5 h, with 45 min of endurance training (10 min of work at the intensity of the ventilatory threshold followed by 5 min of active recovery, repeated 3 times) completed by 30 min of strength-building exercise (8–10 exercises, with sets of 10–15 repetitions). The exercise intensity for the endurance exercise was set individually to the heart rate at the ventilatory threshold [28] and continuously monitored during the session with a cardiofrequency meter. The cycloergometer load was increased over the course of the sessions to maintain the targeted heart rate. The load for the resistance exercise was set at 40% of the isotonic one-repetition maximum (1-RM) of each muscle (deltoid, biceps, triceps and quadriceps) at the first session and progressively increased, targeting 5–6 out of 10 on an exertion scale [29]. This exercise training programme was part of a multi-component and comprehensive pulmonary rehabilitation programme, and it was completed by six small group education sessions (1–1.5 h each) conducted by an experienced therapist specialized in improving health-related behaviours.

### Muscle biopsies

Biopsies from the *vastus lateralis* muscle of the COPD patients and SHS were performed before and after exercise training, using the Bergström technique as previously described [6, 30]. Each biopsy was separated into two samples: one part was immediately frozen in isopentane, precooled in liquid nitrogen, and stored at − 80 °C for histochemical analysis of muscle capillarization (C/F ratio and fibre type characterization; for more details, see Gouzi et al., 2013 [6]); and one part was fixated with a solution of 2.5% glutaraldehyde in PHEM buffer (1X, pH 7.4) and stored at 4 °C for TEM analysis of capillary ultrastructure. The TEM samples were recut into smaller samples (about 12 mm^3^) and immersed in a second fixative solution (0.5% osmic acid) for 2 h in the dark at room temperature. After dehydration (graded series of ethanol, 30–100%), these blocks were embedded in EmBed 812 resin using an Automated Microwave Tissue Processor for Electronic Microscopy (Leica EM AMW) and cut into transversal sections 70 nm thick (Leica-Reichert Ultracut E). These sections were stained with a solution of uranyl acetate and lead citrate and observed with a Tecnai F20 transmission electron microscope at 120 kV (CoMET MRI facilities, INM France) to analyse the capillary ultrastructure.

### TEM image analysis

Quantitative and semi-quantitative analysis of TEM images was performed using ImageJ software. The software was systematically scaled for each image. A mean 12.3 ± 3.2 capillaries/subject for each time (pre- and post-training) was analysed by three independent blinded operators. The reproducibility of the measurement of capillary morphometry between the three operators was assessed. The mean value of the Lin concordance coefficient [31] was 0.98 ± 0.02. The capillary remodelling was assessed using morphometric analysis of the principal capillary components, as previously described [32]. The area (A) and circumference (C) of the capillary structures (Lum, EC, BM, Nucl and Pc) were obtained with the free-hand surrounding tool, as previously described [32]. The thickness (T) of Lum, EC and BM and Lum and the relative volume (RV) of EC, BM and Pc were calculated using formulae proposed by Bigler et al. (2016) [32]: Lum_T_ = 2*A_Lum_/C_Lum_; EC_T_ = 2*[(A_EC_ – A_Lum_ – A_Nucl_)/(C_EC_ + C_Lum_ + C_Nucl_)]; BM_T_ = 2*[(A_BM_ – A_EC_ – A_Pc_)/(C_BM_ + C_EC_ + C_Pc_)]; Lum_RV_ = A_Lum_/A_BM_; EC_RV_ = [(A_EC_ – A_Lum_)/A_BM_]; BM_RV_ = [(A_BM_ – A_EC_)/A_BM_]; Pc_RV_ = A_Pc_/A_BM_. Endothelial nucleus density (ECNuclD) was calculated in the same manner: ECNuclD = [ECNucl_A_/(A_EC_ – Al_um_)]. Morphologic markers of the angiogenic process were assessed according to previous studies [12, 32]. The pericyte coverage (PcCov), considered as the ratio of the lines crossing the BM/EC interface and at least one pericyte to the total lines crossing the BM/EC interface, was obtained using grid-based analysis with test points equal to 0.56 μm^2^. A semi-quantitative analysis was performed to identify the percentage of capillaries associated with Pc/EC interdigitations, abluminal EC sprouts and intraluminal EC folding [9, 12, 32].

### Markers of oxidative stress

The levels of plasma-free and esterified isoprostanes (F_2_-IsoP) were assessed to obtain an index of oxidative stress, as previously reported by Kadiiska et al. [33] and Roberts and Milne (2009). Venous blood samples were collected before and after exercise training and centrifuged (2500 rpm, 4 °C, 10 min) and plasma was stored at − 80 °C until analysis. Free and esterified plasmatic F_2_-IsoP levels were determined using gas chromatography-negative ion chemical ionization mass spectrometry, as previously described [22].

### Statistical analysis

Baseline characteristics of the COPD and SHS groups were compared with t-tests or the Mann-Whitney U depending on the normality and homoscedasticity of the data. Normality was assessed with the Kolmogorov-Smirnov (K-S) test and homoscedasticity with the Brown and Forsythe test [34]. The data are presented as mean (sd) or median (q_1_-q_3_) according to the K-S test result. Correlations are described using the Pearson coefficient. As the two groups (SHS and COPD) were evaluated twice (before and after exercise training), we analysed the data with a linear mixed-effect model, taking into account the repeated measures and using the subject effect as a random effect. For this model, the fixed effects were the Time and Group effects and the interaction between these factors (Group x Time). For variables for which a single parametric value was obtained for each capillary, we performed a multilevel linear mixed-effect model, clustering each capillary value according to its subject of origin to avoid the inflation of Type I error [35]. Linear mixed models were fit using the *lme* function from the *nlme* R package [36]. To further detail our results, we completed the analysis with Fisher’s LSD post-hoc test when the Group x Time interaction term was significant. The normality of the residual distribution was assessed by a Q-Q plot after each *lme* test [37]. Data were analysed with Statistica 7.1 (StatSoft, Inc.) and R 3.5.0 software (www.r-project.org). Data were plotted using GraphPad Prism 5 (GraphPad Software). A *p*-value< 0.05 was considered significant.

## Results

### Baseline values of clinical, functional and histomorphologic parameters and changes in response to exercise training

Among 24 COPD patients and 23 SHS included in the main study [Gouzi et al., 2013] [6], we selected all patients for whom we had at least one muscle sample conditioned for TEM analysis, i.e. 9 COPD patients and 7 SHS. The baseline clinical characteristics of the COPD patients and SHS are presented in Table 1.

**Table 1:** Baseline clinical and functional characteristics of COPD patients and SHS

	COPD	Control	*p*-value
N	9	7	
Sex ratio (male/female)	8 / 1	6 / 1	
Age (years)	57.3 (6.0)	62.6 (4.5)	NS
FEV_1_ (% pred.)	54.6 (17.5)	116.0 (13.1)	*p* < 0.001
FEV_1_ / VC	43.6 (9.6)	76.2 (4.4)	*p* < 0.001
PaO_2_ (mmHg)	71.1 (9.9)		
PA level (Voorips score)	7.3 (3.2–8.3)	4.1 (1.7–4.9)	NS ^$^

Data are presented as mean (SD) or median (Q1-Q3). *FEV*_*1*_ Forced expiratory volume in 1 s, VC slow vital capacity, % pred: % predicted, PaO2 arterial oxygen tension, *PA* Physical activity. ^§^ Mann-Whitney U Test

As shown in Table 2, exercise training induced significant improvement in 6-min walking distance (%pred.) and in endurance time of the quadriceps (Tlim_quad_) in both groups (Time effect: *p* < 0.001 and *p* = 0.009, respectively). As expected, these two functional parameters were significantly lower in COPD patients than in SHS, regardless of the training effect (Group effect: *p* = 0.006 and *p* = 0.024, respectively). A differential effect of exercise training on symptom-limited oxygen uptake ($\mathrm{V}\dot {\mathrm{O}}_{2\mathrm{sl}}\Big)$ was found in COPD patients compared with SHS (Group x Time interaction: *p* = 0.020) with a significant increase in the SHS group only (SHS: 13.8 ± 10.4%, *p* = 0.003 vs COPD: 5.1 ± 8.1%, *p* = 0.28). Regarding the histochemical data, COPD patients showed lower baseline values for the capillary-to-fibre (C/F) ratio compared with SHS (*p* = 0.002 and *p* = 0.024, respectively; Table 2). In response to exercise training, we observed lower improvement in the C/F ratio of COPD patients than SHS (+ 15% versus + 30%; Group x Time interaction: *p* = 0.003), leading to persistent lower post-training values in COPD patients compared with SHS (*p* < 0.001). Similarly, COPD patients presented a reduction of type I fibre proportion in response to exercise training leading to lower post-training values compared to SHS (Group x Time interaction: *p* = 0.01; Additional file 1: Figure. S1).

**Table 2:** Functional and histomorphologic changes in response to exercise training in COPD patients and SHS

	Pre-training		Post-training		p-value
	COPD	SHS	COPD	SHS	
Functional parameters					
$$ \mathrm{V}\dot {\mathrm{O}}_{2\mathrm{sl}} $$(% pred.)	69.3 (17.3) ‡	113.7 (12.7)	72.44 (17.2) ‡	125.6 (9.6) ^###^_$_	T: *p* = 0.004 G: *p* < 0.001 GxT: *p* = 0.020
$$ \mathrm{V}\dot {\mathrm{O}}_{2\mathrm{sl}} $$ (ml/min/kg)	19.8 (4.7) ‡	30.1 (4.4)	21.6 (4.1) ‡	33.1 (7.9) ^##^_$_	T: *p* = 0.009 G: *p* < 0.001 GxT: *p* = 0.046
6MWD (%pred.)	77.2 (10.7)	94.0 (7.3)	84.2 (11.5)	98.7 (7.7)	T: *p* < 0.001 G: *p* = 0.006 GxT: NS
6MWD (m)	539.6 (60.9)	643.3 (60.9)	591.2 (72.4)	684.6 (60.5)	T: *p* < 0.001 G: *p* = 0.010 GxT: NS
Tlim_quad_ (sec)	299.6 (123.4)	588.6 (382.2)	388.6 (201.2) _£_	830.3 (467)	T: *p* = 0.009 G: *p* = 0.024 GxT: NS
Histomorphologic parameters					
C/F ratio	1.29 (0.3) *	1.67 (0.3)	1.49 (0.3) ^##^ ‡	2.18 (0.4) ^###^	T: *p* < 0.001 G: *p* = 0.004 GxT: *p* = 0.003

Data are presented as mean (SD). $$ \mathrm{V}\dot {\mathrm{O}}_{2\mathrm{sl}} $$: symptom-limited oxygen uptake, *6MWD* 6-min walking distance, *Tlim*_*quad*_: endurance time of quadriceps, *C/F ratio* Capillary-to-fibre ratio. _$:_ *n* = 5, _£_: *n* = 8. Linear mixed-effect model, *G* Group effect, *T* Time effect, *GxT* Group x Time interaction. Post-hoc analysis for GxT: different from SHS group at same time: *p < 0.05; † *p* < 0.01; ‡ *p* < 0.001. Different from pre-training: ^##^ *p* < 0.01; ^###^ *p* < 0.001

*Angiogenesis-related capillary remodelling in response to exercise training.*

Capillaries from the *vastus lateralis* muscle biopsies, obtained before and after exercise training in the 9 COPD patients and 7 SHS, were analysed by TEM. The analyses were performed on 392 capillary profiles, which were always composed of at least Lum, EC and BM. Neither total capillary area nor the number of analysed capillaries differed between groups or training times (*p* > 0.05; data not shown). As shown in Table 3, the baseline values of lumen (Lum), endothelium (EC), basement membrane (BM) and pericyte (Pc) relative volume (RV) and thickness (T) did not differ between COPD patients and SHS.

**Table 3:** Baseline morphometric characteristics of capillary components in COPD patients and SHS

	COPD	SHS	p-value
Lum_T_ (nm)	1227 (634)	1203 (522)	0.90
EC_T_ (nm)	388 (122)	358 (116)	0.27
BM_T_ (nm)	211 (85)	220 (75)	0.99
Lum_RV_ (%)	38.2 (16.8)	39.5 (14)	0.53
EC_RV_ (%)	29.6 (9.1)	27.8 (8.3)	0.30
BM_RV_ (%)	21.0 (7)	22.8 (7.4)	0.64
PC_RV_ (%)	5.7 (4.8)	5.9 (4.4)	0.82

*RV* Relative volume, *T* Thickness, *Lum* Lumen, *EC*, Endothelium, *BM* Basement membrane, *Pc* Pericyte. Data are presented as Mean (SD)

**Fig. 1:**
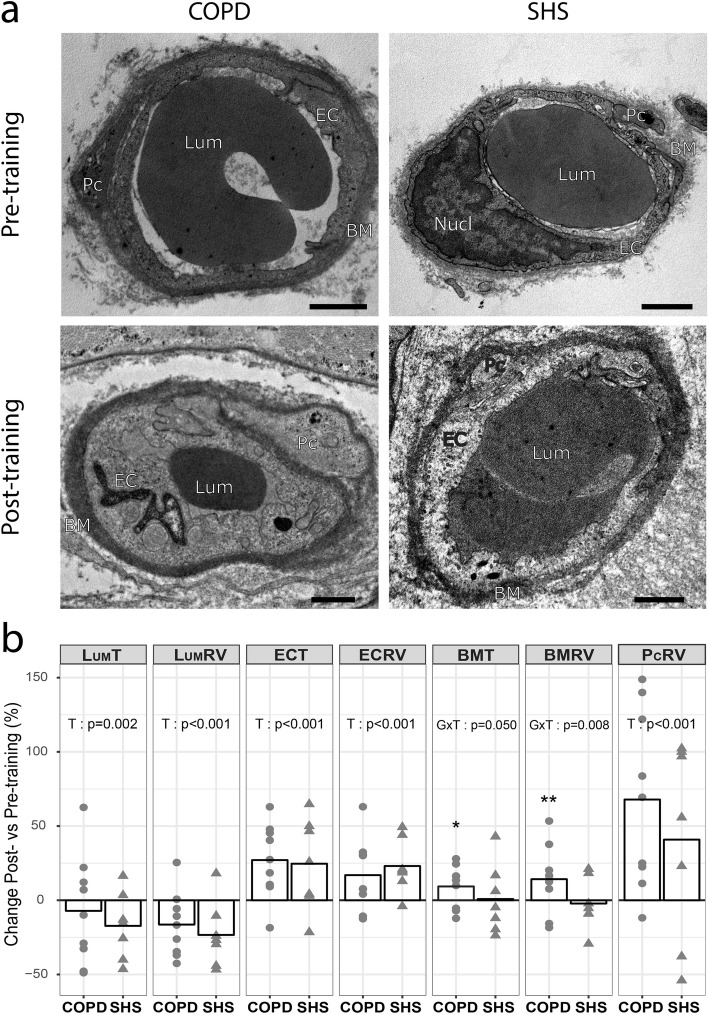
Angiogenesis-related capillary remodelling in response to exercise training in skeletal muscle biopsies of COPD patients and SHS*.* **a** Transmission electron micrographs of capillaries from *vastus lateralis* biopsies of COPD patients and SHS. **b** Quantification of changes in relative volume and thickness of each capillary component following exercise training. RV: relative volume; T: thickness; Lum: lumen; EC: endothelium; BM: basement membrane; Pc: pericyte; Nucl: nucleus. Data are presented as means and individual values. Linear mixed-effect model: T: Time effect; GxT: Group x Time interaction. Post-hoc: Post-training different from Pre-training: *p < 0.05; ** *p* < 0.01. Scale bar: 1 μm

Standard transmission electron micrographs of the skeletal muscle capillaries in COPD patients and SHS, before and after exercise training, are shown on Fig. 1a. Variations of relative volume and thickness of each capillary compartment in response to exercise training are presented in Fig. 1b. In response to exercise training, we observed a significant decrease in Lum_RV_ (COPD: − 16.4 ± 21.5%; SHS: − 23.3 ± 22.1%; Fig. 1b) and Lum_T_ (COPD: − 7.1 ± 36.8%; SHS: − 17.2 ± 22.4%; Fig. 1b) in both groups (Time effect: *p* < 0.001 and *p* = 0.002, respectively). Conversely, a significant training-induced increase was found in both EC_RV_ (COPD: + 17.0 ± 24.1%; SHS: + 22.5 ± 18.5%; Fig. 1b) and EC_T_ (COPD: + 27.1 ± 24.9%; SHS: + 24.7 ± 31.0%; Fig. 1b), regardless of the group (Time effect: *p* < 0.001 for both). Similarly, we observed a significant increase in Pc_RV_ in both the COPD and SHS groups (COPD: + 68.4 ± 60.2%; SHS: + 40.3 ± 65.7%; Time effect: *p* < 0.001; Fig. 1b). Last, a significantly different response to exercise training was observed in COPD patients compared with SHS for BM_RV_ (Group x Time interaction: *p* = 0.008; Fig. 1b) and BM_T_ (Group x Time interaction: *p* = 0.050; Fig. 1b), with a significant increase only in the COPD group for both BM_RV_ (*p* = 0.005; + 14.1 ± 22.6%; SHS: − 2.2 ± 17.3%; Fig. 1b) and BM_T_ (*p* = 0.043; + 9.7 ± 13.8%; SHS: − 0.8 ± 20.2%; Fig. 1b).

*Changes in morphologic markers of the angiogenic process in response to exercise training.*

**Fig. 2:**
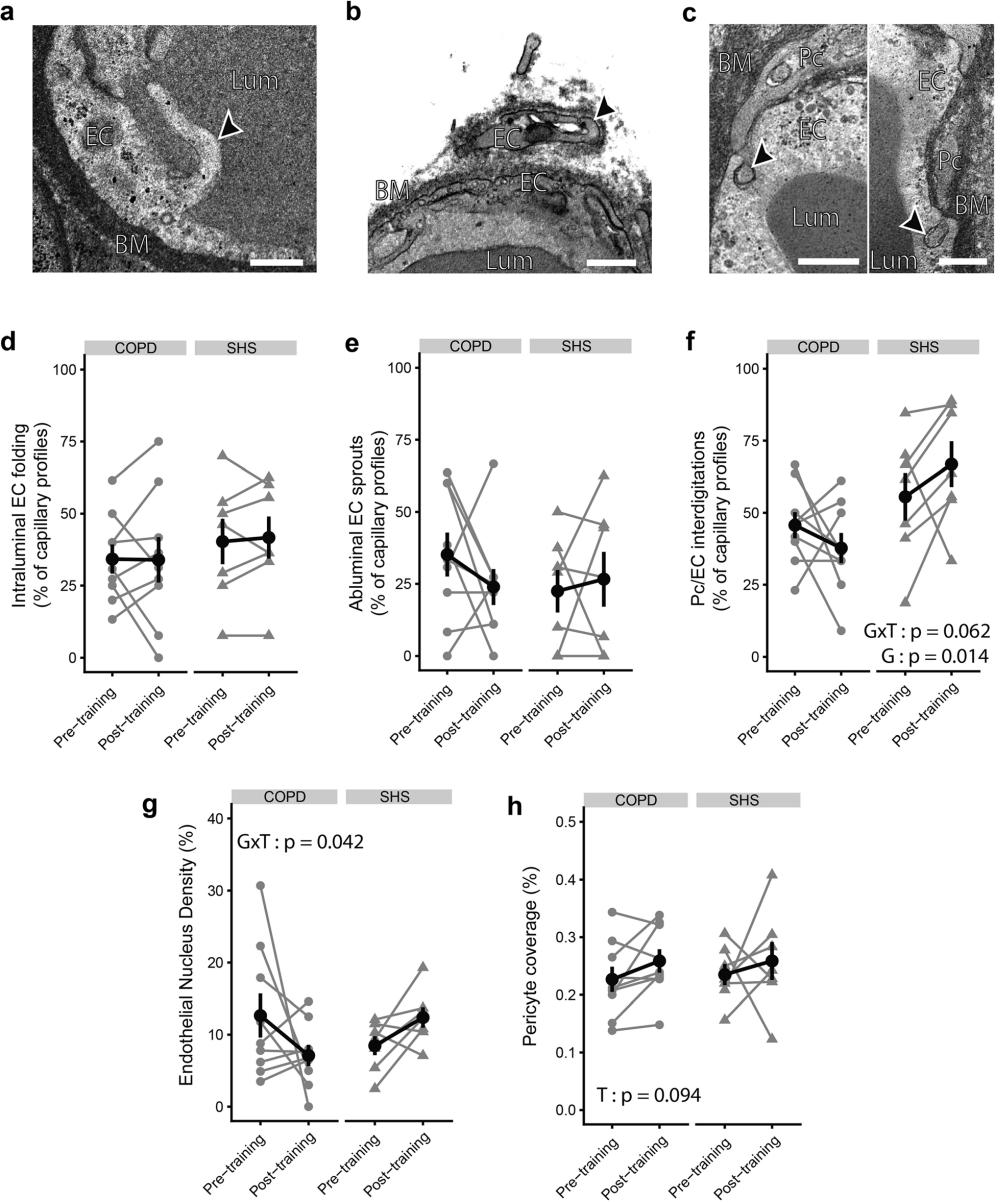
Assessment of morphologic markers of the angiogenic process before and after exercise training in skeletal muscle biopsies of COPD patients and SHS*.* **a-c** Transmission electron micrographs focused on morphologic markers of angiogenesis, shown with black arrows: **a** intraluminal endothelial (EC) folding, **b** abluminal EC sprout, and **c** pericyte/endothelium (Pc/EC) interdigitations. d-f) Semi-quantitative analysis of the occurrence of intraluminal EC folding (**d**), abluminal EC sprouts (**e**), and Pc/EC interdigitations (**f**) on transmission electron micrographs. **g, h** Quantitative analysis of endothelial nucleus density (**g**) and pericyte coverage (**h**) on transmission electron micrographs. Lum: lumen; EC: endothelium; BM: basement membrane; Pc: pericyte. Data are presented as mean ± SE. Linear mixed-effect model: T: Time effect; G: Group effect; GxT: Group x Time interaction. Scale bar: 500 nm

We quantified the ultrastructural morphologic markers of angiogenesis, which are presented on the transmission electron micrographs in Fig. 2a-c. As shown in Fig. 2d and e, the occurrence of intraluminal EC folding or abluminal EC sprouts did not increase significantly with exercise training (*p* > 0.05). COPD patients and SHS did not show different pre- or post-training values for these indexes either (*p* > 0.05). Conversely, Pc/EC interdigitation occurrence was significantly lower in the COPD group than in SHS (Fig. 2f) (pre-training: 0.46 ± 0.14% vs 0.56 ± 0.22%; post-training: 0.38 ± 0.16% vs 0.67 ± 0.21%; Group effect: *p* = 0.014). Moreover, taking into account the baseline difference between COPD patients and SHS, we noted a tendency towards a differential effect of exercise training on this parameter between the two groups (Group x Time interaction: *p* = 0.062). Similarly, we observed significantly different variations of endothelial nucleus density (ECNuclD) in the capillaries of COPD patients compared with SHS in response to exercise training (Group x Time interaction: *p* = 0.042; Fig. 2g). ECNuclD tended to decrease in response to exercise training only in the COPD patients (− 7.3 ± 67.1%, *p* = 0.053; Fig. 2g), leading to a tendency towards lower post-training ECNuclD values in the patients compared with SHS (*p* = 0.080; Fig. 2g). The pericyte coverage (PcCov) tended to increase significantly with exercise training in both groups (Time effect: *p* = 0.094; Fig. 2h), with no difference between the groups (COPD: + 17.6 ± 26.1%; SHS: + 16.9 ± 50.5%).

**Fig. 3:**
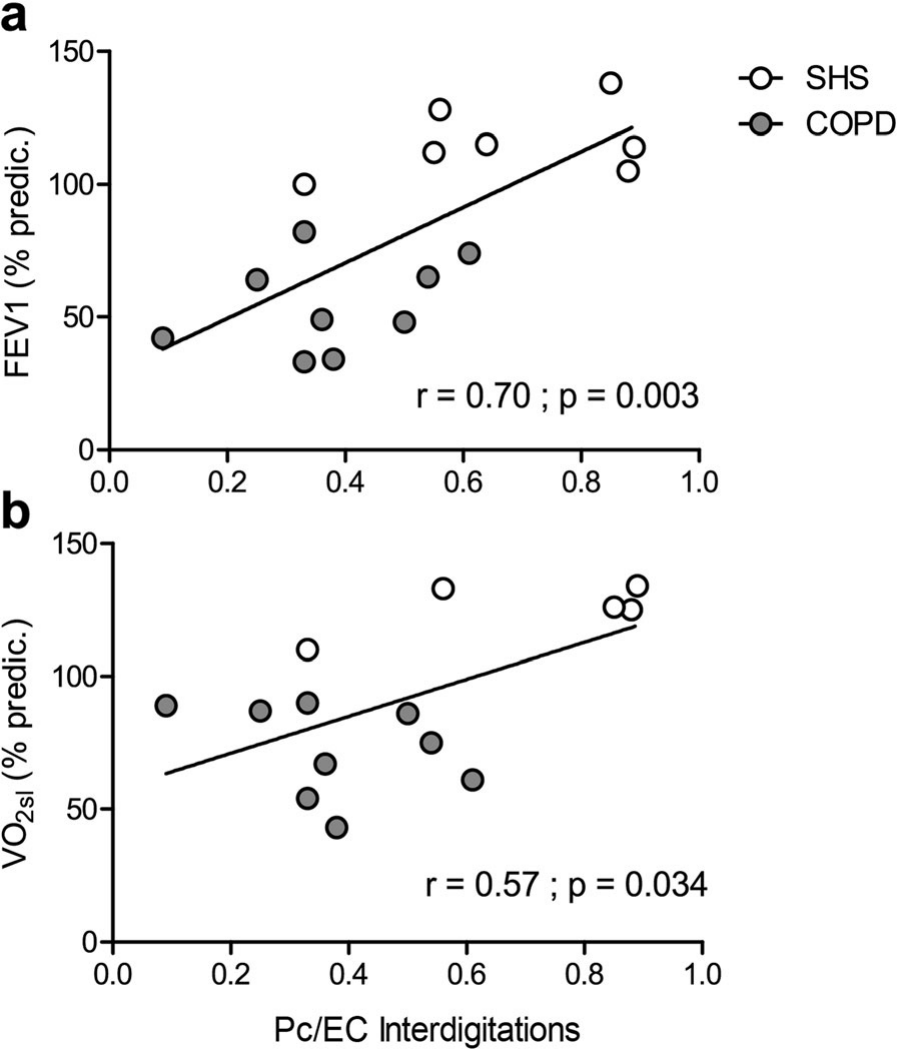
Correlations between morphologic markers of the angiogenic process and clinical and functional parameters of COPD patients and SH*S.* Positive significant correlations between the proportion of capillary profiles associated with Pc/EC interdigitations in post-training *vastus lateralis* biopsies and **a** forced expiratory volume in 1 s (FEV_1_) (% predic.), **b** post-training values of symptom-limited oxygen uptake ($\mathrm{V}\dot {\mathrm{O}}_{2\mathrm{sl}}$), in both COPD patients (grey circles) and SHS (white circles)

Correlations between the morphologic markers of the angiogenic process and the clinical, functional and histochemical parameters of COPD patients and SHS are presented in Fig. 3. After the 6-week exercise training programme, we found significant and positive correlations between the occurrence of Pc/EC interdigitations and FEV_1_ (%pred.; r = 0.70, *p* = 0.003; Fig. 3a), $\mathrm{V}\dot {\mathrm{O}}_{2\mathrm{sl}}$ (%pred.; r = 0.57, *p* = 0.034; Fig. 3b).

### Training-induced changes in plasmatic F_2_-IsoP levels

**Fig. 4:**
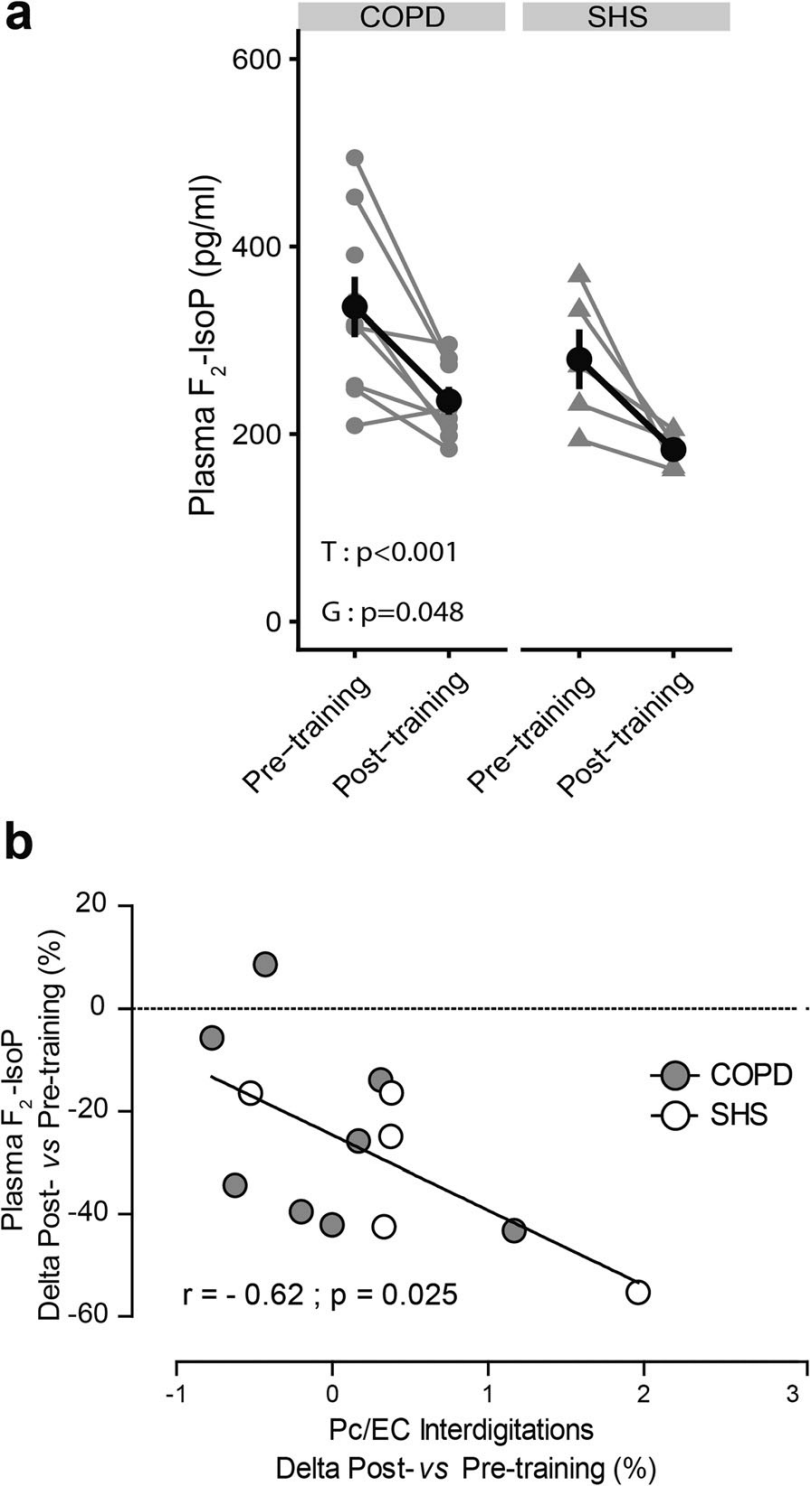
*Changes in plasma-free and esterified isoprostane* (*F*_*2*_*-IsoP) levels in response to exercise training in COPD patients and SHS and the relation to the angiogenic process.* **a** Effect of exercise training on plasmatic levels of F_2_-IsoP in COPD and SHS. Linear mixed-effect model: T: Time effect, G: Group effect. Pre-training: COPD: *n* = 9, SHS: *n* = 5; Post-training: COPD: *n* = 8, SHS: *n* = 6. Data are presented as Mean ± SE. **b** Correlation between the pre-to-post training variations of plasmatic F_2_-IsoP levels and the pre-to-post training variations in Pc/EC interdigitation occurrence in COPD patients (grey circles) and SHS (white circles)

As shown in Fig. 4a, plasmatic levels of F_2_-IsoP decreased significantly with exercise training in both COPD patients and SHS (− 24.5 ± 19.1% and − 31.1 ± 17.2%, respectively; Time effect: *p* < 0.001). However, regardless of exercise training, plasma F_2_-IsoP was significantly higher in COPD patients than in SHS (pre-training: 335.7 ± 96.1 vs 280.0 ± 71.4; post-training: 235.6 ± 42.2 vs 183.5 ± 16.9; Group effect: *p* = 0.048; Fig. 4a). Moreover, as shown in Fig. 4b, we found a significant and negative correlation between the changes in Pc/EC interdigitations and the changes in plasmatic levels of F_2_-IsoP in response to exercise training (r = − 0.62, *p* = 0.025).

## Discussion

This study is the first to assess skeletal muscle capillary ultrastructure after exercise training-related angiogenesis in COPD patients. The main result is that the blunted training-induced angiogenesis previously found in COPD patients [6] is associated with abnormal capillary remodelling and morphologic features of an impaired angiogenic process.

Consistent with the previous study in this trial, COPD patients showed significant but blunted improvements in exercise capacity and muscle angiogenesis (Table 2) and an impaired adaptation of the muscle typology (Additional file 1: Figure S1) in response to exercise training in comparison with sedentary healthy subjects. Together, these observations highlight an impairment of the oxidative metabolism of COPD patients and a misadaptation of this last to exercise training. However, the changes in fibre type under exercise training and the training-induced angiogenesis were poorly correlated (Additional file 1: Figure S1). Moreover, another study on COPD patients found differential angiogenic responses to exercise training between two groups of COPD patients, although the groups presented similar adaptations of skeletal muscle typology to the training [38]. Muscle typology alone was thus insufficient to explain the blunted angiogenesis found in these COPD patients, which led us to suspect that other cellular mechanisms might be involved. We therefore conducted this study to investigate capillary remodelling and the morphologic markers of the angiogenic process associated with the blunted training-induced angiogenesis found in these patients. The capillary remodelling and the morphologic markers of the angiogenic process were assessed blindly following cross-validated and previously published methods [12, 32]. The inter-observer reliability of the measurements of capillary remodelling was assessed with three observers and was “excellent” (according to Lin’s concordance coefficient) [39].

Our study brings the first evidence of an exercise training effect on capillary ultrastructure in both COPD patients and healthy subjects between 50 and 70 years old and identified as strictly sedentary using a validated method. Both exercise training and ageing are known to alter the capillary ultrastructure in skeletal muscles [12, 32]. To isolate the impact of COPD on capillary ultrastructure and its response to exercise training, we selected COPD patients and SHS who did not differ in age or physical activity level for this study. In SHS, we observed a significant increase in both EC_RV_ and EC_T_ and no changes in BM_RV_ and BM_T_ in response to the training (Fig. 1b,c), as previous described in younger and more active healthy subjects [12, 40]. In contrast, we found a decrease in both Lum_RV_ and Lum_T_ in these SHS following the training programme (Fig. 1b,c), and this decrease has been described as part of the angiogenic process in skeletal muscle [9, 10]. Physiologically, angiogenesis occurs in two phases, an early phase of capillary creation (by sprouting and/or intussusception) in the first 5 weeks of exercise training and then a late phase of capillary maturation [7, 41]. Consistent with this time-course, we observed later angiogenesis-related changes (increased PcCov and PC_RV_) in SHS at 6 weeks of exercise training (Fig. 1b and Fig. 2f,h) but not the early angiogenesis hallmarks (abluminal EC sprouts or intraluminal EC folding; Fig. 2d,e), in line with the findings of an earlier study in younger healthy subjects [12].

Conversely, the COPD patients presented an abnormal remodelling of capillaries in response to exercise training. While their adaptation of lumen (Lum_RV_ and Lum_T_) and endothelium (EC_RV_ and EC_T_) in response to training was physiological, they showed an unexpected increase in BM_RV_ and BM_T_ (Fig. 1b,c). The BM thickening after training was a non-physiological response, and this original result contrasts with what has been shown in other chronic diseases like hypertension, in which training induced BM thinning [40]. Exercise-induced oxidative stress [42], which is a hallmark of COPD, may be a mechanism in this modification, because oxidative stress disturbs the BM turnover in favour of an accumulation of the BM component [16]. Last, the loss of Pc/EC interdigitations (Fig. 2f) may also have contributed to the BM thickening. Indeed, these interdigitations act as a mechanical link between the endothelium and pericytes, helping the capillaries to resist the dramatic increase in haemodynamic forces during exercise [43, 44]. Thus, the loss of the pericyte-endothelium link in COPD patients could be the cause of compensatory BM thickening.

In addition, our study showed an impaired angiogenic process in response to exercise training in the COPD patients. The skeletal muscle angiogenic process of COPD patients and SHS in response to exercise training was assessed through TEM analysis of the morphologic markers of specific capillary alterations [8–10, 12]. These markers inform about angiogenesis-related mechanisms and possible impairments [9, 12]. As described above, our measures were performed too late to assess the morphologic markers of the early phase of angiogenesis [7] (i.e. abluminal EC sprouts and intraluminal EC folding), and we thus found no changes in these markers in the COPD patients (Fig. 2d,e). However, it is possible to assess the final result of capillary proliferation by measuring the nucleus density in endothelial cells [8, 12]. Here, we found a significantly different response to exercise training for the ECNuclD in the COPD patients compared with that of healthy subjects (Fig. 2g), suggesting impaired capillary proliferation during the angiogenic process. This finding is consistent with the blunted angiogenesis found at the histomorphologic level in these patients (Table 2). After 6 weeks of exercise training, we assessed the later phase of the angiogenic process, capillary maturation [7]. The Pc/EC interdigitations, as observed by TEM, are defined as the morphologic expression of capillary maturation [11], which increases during skeletal muscle stimulation [9]. In our study, we found a tendency towards a differential variation in the occurrence of the Pc/EC interdigitations in the COPD patients compared with SHS (Fig. 2f). Moreover, independently of exercise training, the patients presented a significantly lower occurrence of Pc/EC interdigitations. Surprisingly, the PcCov was not impaired in the COPD patients. However, while PcCov is described as a marker of capillary maturation, the maturation effect of pericyte is mainly driven by the exchange of multiple angiogenic factors. Among these, Ang1 plays a crucial role [45] and its exchange mostly occurs at the level of Pc/EC interdigitations [46]. Thus, while the PcCov in COPD patients was not impaired, the loss of Pc/EC interdigitations could lead to a dysfunctional pericyte coverage. However, both Ang1/Tie2 axis and pericyte function remains to be experimentally assessed in skeletal muscle capillaries of COPD patients. With these results, we bring the first evidence of impaired capillary maturation during exercise training-related angiogenesis in COPD patients. Regardless of exercise training, the COPD patients in our study presented higher levels of plasmatic F_2_-IsoP, a marker of oxidative stress-related lipid peroxidation [18], than SHS (Fig. 4a). In addition, the changes in the occurrence of Pc/EC interdigitations in response to the training were negatively correlated with the changes in the plasmatic level of F_2_-IsoP (Fig. 4b). In the literature, oxidative stress has been experimentally related to pericyte loss [17, 47]. Indeed, it can increase angiopoeitin-2 levels [48], which antagonize the interaction between endothelial cells and pericytes [49, 50]. Moreover, partial suppression of F_2_-IsoP by antioxidant supplementation prevents pericyte loss [20]. Based on the literature and the F_2_-IsoP results of the present study, we hypothesize that oxidative stress can contribute to the impairment of capillary maturation found in COPD patients. Further experimental studies with oxidative stress intervention (pro- and anti-oxidant treatment in combination with training) in an animal model and COPD patients, associated with TEM analysis, are required to confirm this hypothesis.

The present study shows impairments in capillary remodelling and the angiogenic process in response to exercise training in COPD patients. As discussed in the previous paragraphs, our results constitute an original contribution to knowledge on the muscle cellular adaptations to cardiopulmonary rehabilitation. This study may be of interest to researchers working in the field of skeletal muscle dysfunction and angiogenesis in chronic respiratory diseases because it provides evidence that can guide their research questions and it specifically targets the angiogenesis mechanisms, i.e. the Pc/EC interactions. Our study constitutes a step forward in the search for complementary therapeutic interventions to restore physiological muscle adaptations to exercise training in COPD patients. While it was not the aim of our study, our results raise the question of their clinical relevance. Indeed, BM thickening increases the width of the capillary wall and thus the distance between erythrocytes and mitochondria. Because this distance is crucial in the process of oxygen diffusion and supply to the exercised muscle [51], this BM thickening might limit the improvements in muscle oxygen consumption and $\mathrm{V}\dot {\mathrm{O}}_{2\mathrm{sl}}$ in patients, as in peripheral artery disease patients [13]. Also, contractile pericytes [43] can directly regulate capillary blood flow [52]. Thus, the loss of the Pc/EC interdigitations in COPD patients might also impact the oxygen supply [53] and $\mathrm{V}\dot {\mathrm{O}}_{2\mathrm{sl}}$. Consistently, we found a positive correlation between post-training Pc/EC interdigitation occurrence and $\mathrm{V}\dot {\mathrm{O}}_{2\mathrm{sl}}$ (Fig. 3b). Last, given that capillary maturation prevents the regression of neo-capillaries [54, 55], the impaired capillary maturation might constitute a mechanism for the blunted training-related angiogenesis in COPD patients. Specifically designed studies with larger samples are required to generalize these results to the whole COPD population and to establish the clinical implications of the defective capillary remodelling and angiogenic process in these patients, as reported in our exploratory study.

## Conclusion

Exercise training improves muscle function in COPD patients but to a lesser extent than in healthy subjects, especially regarding the increase in muscle capillarization (i.e. angiogenesis). Here, we provide the first evidence that this blunted angiogenesis is characterized by abnormal capillary remodelling and an impairment in both phases of the angiogenic process, i.e. capillary creation and maturation, in response to exercise training. The loss of pericyte-endothelium interaction appears to be a key factor for the capillary maturation. This study contributes to our understanding of the defective angiogenic process in COPD patients and constitutes a significant step towards isolating the blunted cellular mechanisms in endothelial cells and pericytes. Our observations suggest a hypothetic role for oxidative stress in the angiogenesis defect and open the way for further studies investigating this relationship in COPD patients.

## Supplementary information

**Additional file 1 Figure S1**. *Changes in skeletal muscle typology in response to exercise training in COPD patients and SHS and the relation to the angiogenic process*. a) Effect of exercise training on the proportion of type I fibre in *vastus lateralis* cryosections of COPD patients (grey bars) and SHS (white bars). Linear mixed-effect model: GxT: Group x Time interaction. Post-hoc: *p < 0.05; *** *p* < 0.001. Data are presented as mean ± SE. b) Correlation between the post-to-pre training variations of type I proportion and the post-to-pre training variations of capillary-to-fibre ratio in COPD patients (grey circles) and SHS (white circles).

## Data Availability

The datasets used and/or analysed during the current study are available from the corresponding author on reasonable request.

## Abbreviations

A: Area
BM: Basement Membrane
C: Circumference
C/F: Capillary-to-fibre
COPD: Chronic obstructive pulmonary disease
EC: Endothelial
ECNuclD: Endothelial nucleus density
F2-IsoP: Plasma-free and esterified isoprostane
FEV1: Forced expiratory volume in one second
Lum: Lumen
Pc: Pericyte
PcCov: Pericyte coverage
RV: Relative volume
SHS: Sedentary healthy subjects
T: Thickness
TEM: Transmission electron microscopy
Tlimquad: Endurance time of the quadriceps
V̇O2sl: Symptom-limited oxygen uptake
