# Supplementary figures and images for: Impaired training-induced angiogenesis process with loss of pericyte-endothelium interactions is associated with an abnormal capillary remodelling in the skeletal muscle of COPD patients
Source: Respir Res. 2019 Dec 5;20:278. doi: 10.1186/s12931-019-1240-6 (PMC6896673; doi:10.1186/s12931-019-1240-6)

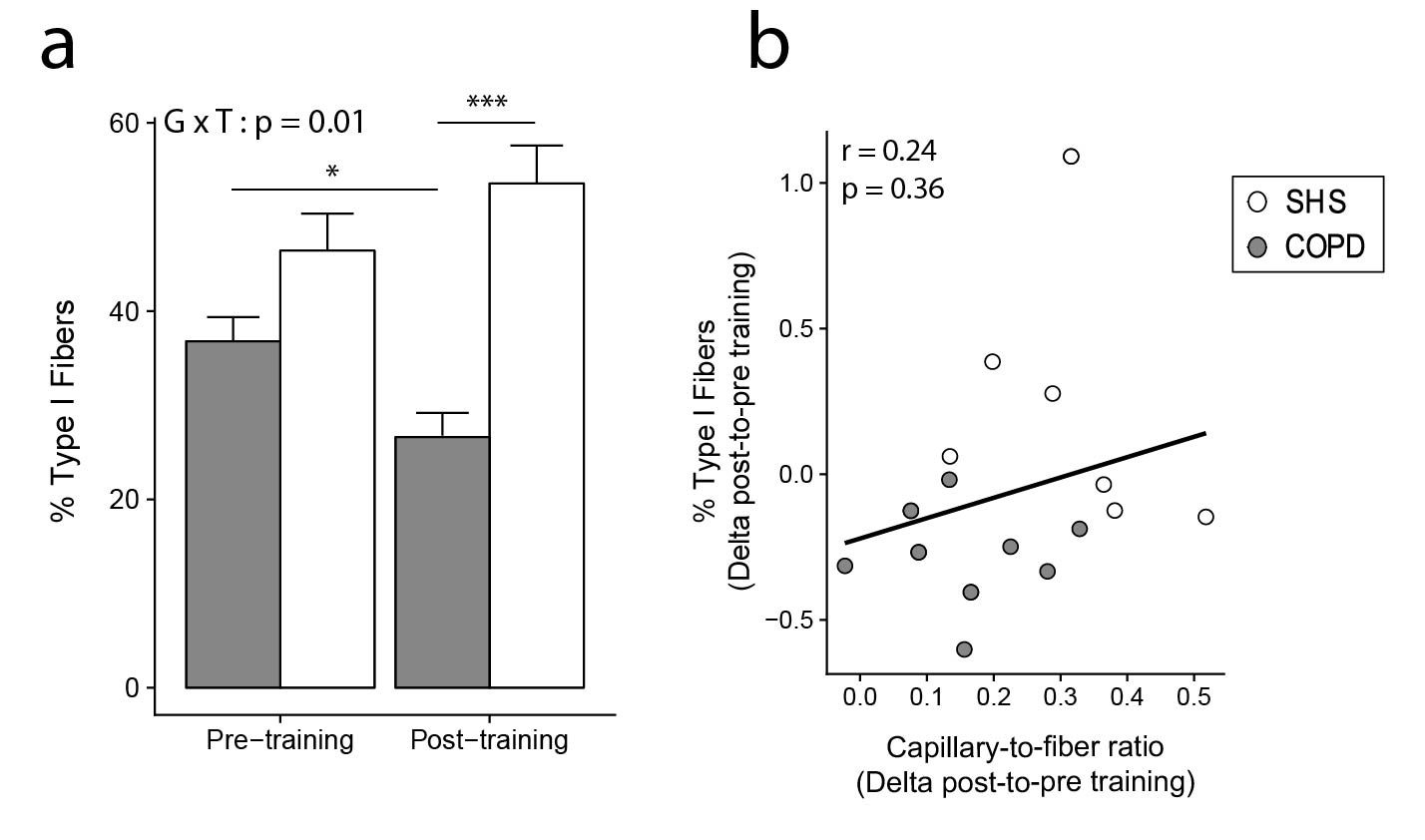

Supplement: Supplementary file 1 — Additional file 1 Figure S1. Changes in skeletal muscle typology in response to exercise training in COPD patients and SHS and the relation to the angiogenic process. a) Effect of exercise training on the proportion of type I fibre in vastus lateralis cryosections of COPD patients (grey bars) and SHS (white bars). Linear mixed-effect model: GxT: Group x Time interaction. Post-hoc: *p < 0.05; *** p < 0.001. Data are presented as mean ± SE. b) Correlation between the post-to-pre training variations of type I proportion and the post-to-pre training variations of capillary-to-fibre ratio in COPD patients (grey circles) and SHS (white circles). [file 12931_2019_1240_MOESM1_ESM.jpg]
